# Clinical Features and Outcome of Mucormycosis

**DOI:** 10.1155/2014/562610

**Published:** 2014-08-20

**Authors:** Carlos Rodrigo Camara-Lemarroy, Emmanuel Irineo González-Moreno, René Rodríguez-Gutiérrez, Erick Joel Rendón-Ramírez, Ana Sofía Ayala-Cortés, Martha Lizeth Fraga-Hernández, Laura García-Labastida, Dionicio Ángel Galarza-Delgado

**Affiliations:** ^1^Departamento de Medicina Interna, Hospital Universitario “Dr. José E. González”, Universidad Autónoma de Nuevo León, Madero y Gonzalitos S/N, 64460 Monterrey, NL, Mexico; ^2^Servicio de Anatomia Patologica, Hospital Universitario “Dr. José E. González”, Universidad Autónoma de Nuevo León, Madero y Gonzalitos S/N, 64460 Monterrey, NL, Mexico

## Abstract

Mucormycosis (MCM) is a life-threatening infection that carries high mortality rates despite recent advances in its diagnosis and treatment. The objective was to report 14 cases of mucormycosis infection and review the relevant literature. We retrospectively analyzed the demographic and clinical data of 14 consecutive patients that presented with MCM in a tertiary-care teaching hospital in northern Mexico. The mean age of the patients was 39.9 (range 5–65). Nine of the patients were male. Ten patients had diabetes mellitus as the underlying disease, and 6 patients had a hematological malignancy (acute leukemia). Of the diabetic patients, 3 had chronic renal failure and 4 presented with diabetic ketoacidosis. All patients had rhinocerebral involvement. In-hospital mortality was 50%. All patients received medical therapy with polyene antifungals and 11 patients underwent surgical therapy. Survivors were significantly younger and less likely to have diabetes than nonsurvivors, and had higher levels of serum albumin on admission. The clinical outcome of patients with MCM is poor. Uncontrolled diabetes and age are negative prognostic factors.

## 1. Introduction 

Mucormycosis (MCM) is a devastating infection with high mortality rates despite recent advances in its diagnosis and treatment. It is caused by the filamentous fungi of the Mucorales order of the class of Zygomycetes [1]. Although it is classically defined as an opportunistic infection, preferentially affecting patients with diabetes mellitus (DM), neutropenia, malignancy, chronic renal failure, and acquired immunodeficiency syndrome and those who have received organ or hematopoietic stem cell transplants, it can affect immunocompetent hosts as well (such as trauma patients) [1, 2]. The incidence of MCM worldwide appears to be increasing, particularly in oncological patients and those with DM [3]. Along with aspergillus, it is one of the most common invasive fungal infections affecting immunosuppressed individuals.

Despite aggressive surgical and polyene antifungal therapy, overall mortality for MCM infection remains high, with figures ranging from 20 to 50% [4–6]. Depending on patient characteristics (such as critically ill or immunocompromised patients) and site of infection, mortality rises markedly, nearing 70–90% for cases of disseminated mucormycosis [4–6]. Inhalation of sporangiospores is the most common route of transmission, although ingestion of spores, direct implantation into injured skin (burns), trauma with contaminated soil, or intravenous (drug users) transmission have also been described [7]. After nasal inoculation it takes a rapidly progressive course extending to neighboring tissues, including the orbit, and sometimes to the brain. Lipid formulations of amphotericin B are the mainstay of treatment, along with aggressive surgical therapy [8]. However, such drug formulations are not available worldwide due to their elevated costs.

Most of the information on the epidemiology and clinical characteristics of MCM comes from case series and small studies on specific populations, such as those in oncology centers. Considering the high mortality associated with MCM and the increasing recognition of the importance of this disease in Latin American countries, we set out to describe the demographic and clinical characteristics of MCM patients in a tertiary-care teaching hospital in Mexico.

## 2. Materials and Methods

### 2.1. Patients

We received approval from the ethics committee of our institution to carry out this study. From 2007 to 2012, we identified all patients with MCM at the University Hospital “Jose Eleuterio Gonzalez,” and their records were obtained to analyze the cases retrospectively. Demographic characteristics, presentation, signs, and symptoms as well as treatment and outcomes were analyzed. Cases were sought through manual and electronic searches in hospital records (using discharge diagnoses). All patients had been hospitalized (none came from outpatient clinics).

### 2.2. MCM Diagnostic Criteria

We used the 2008 European Organization for Research and Treatment of Cancer/Mycoses Study Group (EORTC/MSG) criteria for the diagnosis of MCM [9], but only proven cases were retained. All cases had expert histopathological confirmation and were culture positive. Samples were most commonly obtained from the sinus cavities.

### 2.3. Statistical Methods

Descriptive statistical methods were used to analyze demographic characteristics, and differences between groups were calculated using Student's  *t*-test for independent samples or Fisher's exact test, where appropriate. Statistical significance was established at *P* < 0.05. Data were analyzed using an SPSS statistical package (SPSS Statistics 15.0, SPSS Inc., Armonk, NY).

## 3. Results

### 3.1. Patient Characteristics

We found 14 patients meeting our search criteria.* Rhizopus* was identified as the genus in all cases. The demographic characteristics of the patients are reported in Table 1. The mean age of the patients was 39.9 +/− 20.3 years (range 5–65 years), with men presenting the majority (64.3%). The most common underlying disease was DM (71.4%), followed by hematological malignancy (42.8%) and chronic renal insufficiency on hemodialysis (21.4%). All patients with chronic renal insufficiency were diabetic. Five patients with hematological malignancy had ALL and only one had acute myelocytic leukemia; they had all received chemotherapy, including steroids, and two were neutropenic on diagnosis. Of the diabetic patients, 4 presented with diabetic ketoacidosis. Hypertension was a common comorbidity (50%). Three of the patients were children, all with ALL.

### 3.2. Presentation

Presenting signs and symptoms are reported in Table 2. Fever (71.4%) and rhinorrhea (57.1%) were the most common signs. Patients also complained of headache, ocular pain, facial edema, and when ocular involvement was present, visual abnormalities.

### 3.3. Sites of Infection and Treatment

**Figure 1:**
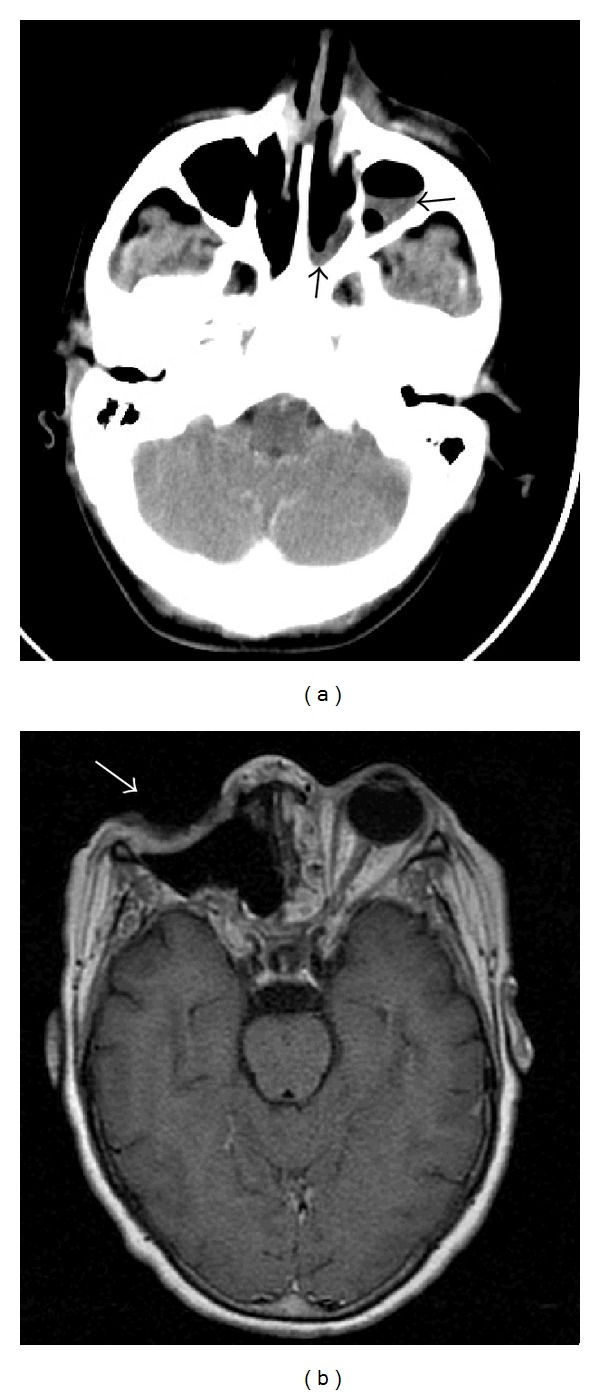
(a) An example of a computed tomography showing sinusitis (black arrows). Biopsies were positive for mucormycosis in this patient. (b) Postsurgical magnetic resonance image (T1 weighted) in a patient with ocular involvement. The patient recovered well.

All patients had rhinocerebral involvement (Figure 1). On presentation, 4 patients had overt orbital involvement. All patients received medical treatment with conventional (deoxycholate) amphotericin B at 1–1.5 mg/kg body weight/day within 24 hours of admission. Amphotericin B was administered through a central line, with a median duration of treatment of 11.5 days. Medical treatment was initiated empirically in 10 patients and after microbiological identification in the remaining cases. Hypokalemia and creatinine elevations appeared in 6 patients after treatment, but these alterations did not ultimately require switching or modifying therapy. Surgery was performed in 11 (78.6%) of cases (Figure 1). We did not identify cases of disseminated MCM.

### 3.4. Outcome

In-hospital mortality was 50%. Mean hospital stay was 19.5 days (range: 1–79 days). The stratified characteristics of survivors and nonsurvivors are reported in Table 1. Nonsurvivors were older and more likely to be diabetic. All of the patients that presented with diabetic ketoacidosis died, and all nonsurvivors underwent surgery. Some routine laboratory tests on admission were also analyzed. No differences were found in hemoglobin, white blood count, or serum electrolytes (data not shown) between survivors and nonsurvivors. Mean creatinine was 3.68 +/− 3.15 mg/dL in nonsurvivors and 1.69 +/− 1.15 mg/dL in survivors, with no statistical difference between groups. Serum albumin levels were significantly lower on admission in nonsurvivors (2.3 +/− 0.23 g/dL) in comparison with survivors (3.15 +/− 0.46 g/dL), with a *P* = 0.03. There was no difference in the time of initiation of treatment between survivors and nonsurvivors (data not shown).

## 4. Discussion

The majority of the patients in our series were male, a trend that has been consistently reported in case series from different countries [10–12]. The most common underlying disease in our series was DM. DM is associated with impaired neutrophil function, microvascular insufficiency, and in the case of ketoacidosis, other metabolic abnormalities that promote fungal growth [11–13].* Rhizopus* species have an active ketone reductase system and thrive in high glucose and acidotic conditions. These patients also have decreased phagocytic activity because of impaired glutathione pathway. Normal serum inhibits Rhizopus whereas serum of the diabetic ketoacidosis patients stimulates its growth [14]. Notably, in our series, 4 patients presented with diabetic ketoacidosis. In a series of 28 MCM cases, 64% cases had DM and 55.6% of those cases had diabetic ketoacidosis [15], a proportion similar to our findings. However, in other series, complications associated with DM accounted for only 17% of cases of MCM [16]. Chronic renal insufficiency is another condition that predisposes to MCM infection. In our series, patients with renal disease all had concomitant DM and were on hemodialysis. In a 1997 case series with patients from our institution, rhinocerebral MCM was diagnosed in 22 patients over 15 years [17]. Twenty of these patients were diabetic, and half presented with ketoacidosis. In contrast with our study, hematologic malignancy was uncommon, with only one patient presenting with myelodysplastic syndrome [17]. This could indicate increasing number of patients with hematological malignancies admitted to our institution, the use of more aggressive immunosuppressive regimens in these patients, and improved control of DM complications in our population. These trends have been recognized in other studies as well [12, 16].

In the largest case series, patients with hematological malignancies (mainly acute leukemia) represent the group with the highest prevalence and with rapidly increasing rates of MCM [12, 16]. In our series 6 of our patients had hematological malignancies, and in all cases these were acute leukemia (ALL and AML). Although neutropenia has also been hailed as an important factor in the development of MCM, only 2 of our patients with ALL presented with neutropenia.

MCM infection may have a rhinocerebral, rhinoorbital, pulmonary and soft-tissue extension, among others. Moreover, it can also present as a devastating disseminated form. All cases in our series had rhinocerebral or rhinoorbital involvement. Recent registries have reported conflicting data about the most common site of infection. In a global registry, the lung (58.5%) was the main site of infection, followed by rhinocerebral or rhinoorbital involvement (19.5%) [16]. In an Italian series, rhino-orbital-cerebral involvement was the most common site of infection (35%), followed by the lung (25%) [18]. Interestingly, we found no evidence of lung involvement or of disseminated disease in our series.

The most common signs and symptoms were fever, rhinorrhea, and headache, while the most ominous symptom was vision loss. Despite aggressive medical and surgical treatment, in-hospital mortality in our patients was 50%, which is similar to rates reported in other case series [16]. Although it is higher than the rate reported in a recent population-wide study (22%) [4], our series is quite different in terms of infection site (mainly rhinocerebral) and treatment (conventional amphotericin B). All of our patients received conventional (as opposed to liposomal) amphotericin B at doses that ranged from 1 to 1.5 mg/kg/day. Although surgical therapy has been associated with improved outcome in some studies [5, 6], all of the nonsurvivors in our study underwent surgical treatment. This could be explained by clinically milder forms of MCM infection in those patients that did not have to undergo surgery.

Notwithstanding our small sample, we could identify factors that were significantly different in nonsurvivors when compared with survivors: these were older age, DM, and ketoacidosis at presentation. Ketoacidosis represents a severe complication of DM in its own right and indicates poor glycemic control, so along with old age it is not surprising that it would be associated with worse outcomes. Of note, all of the patients with chronic renal failure died as well. This accounts for the higher creatinine levels (although not significant) on admission in nonsurvivors. Serum albumin on admission was lower in nonsurvivors as well, which could indicate malnutrition, another predisposing factor for immunosuppression.

## 5. Conclusion

In conclusion, MCM is a life-threatening infection that most commonly affects immunocompromised individuals and that despite aggressive multimodal treatment carries a significant risk of mortality. A high index of suspicion is required in order to begin the appropriate diagnostic workup and treatment. Our cases most commonly involved the rhino-orbital-cerebral cavities, and the main underlying disease was DM. Unfortunately, due to economic limitations, the use of liposomal amphotericin B in third world countries is often prohibitive, and our patients were instead treated with conventional amphotericin B. Fortunately, there were no cases in our series where side effects (such as renal injury or hypokalemia) forced a change in therapy. In light of evidence suggesting that early and aggressive use of liposomal amphotericin B could improve outcomes [18], this issue should be evaluated thoroughly.

## Figures and Tables

**Table 1:** Demographic data.

	Total (%)	Survivors (%)	Nonsurvivors (%)	*P*
Patients	14 (100)	7 (100)	7 (100)	
Female	5 (35.7)	2 (28.6)	3 (57.1)	0.4
Male	9 (64.3)	5 (71.4)	4 (57.1)	0.5
Age (years)	39.9	27.4	52.4	**0.03** ∗
DM	10 (71.4)	3 (42.8)	7 (100)	**0.03** ∗
HM	6 (42.8)	4 (57.1)	2 (28.6)	0.29
CRI	3 (21.4)	0 (0)	3 (42.8)	0.09
KET	4 (28.6)	0 (0)	4 (57.1)	**0.03** ∗
Hypertension	7 (50)	2 (28.6)	5 (71.4)	0.14
Surgery	11 (78.6)	4 (57.1)	7 (100)	0.09

CRI: chronic renal insufficiency; DM: diabetes mellitus; HM: hematological malignancy; KET: diabetic ketoacidosis. Significant *P* values are indicated in bold.

*P* ≤ 0.05.

**Table 2:** Presenting signs and symptoms.

Sign/symptom	*n* (%)
Fever	10 (71.4)
Rhinorrhea	8 (57.1)
Cephalea	7 (50)
Ocular pain	4 (28.6)
Vision loss	3 (21.4)
Palpebral edema	3 (21.4)
Facial edema	2 (14.3)
Proptosis	1 (7.1)

## References

[1] Spellberg B, Walsh TJ, Kontoyiannis DP, Edwards JJ, Ibrahim AS (2009). Recent advances in the management of mucormycosis: from bench to bedside. *Clinical Infectious Diseases*.

[2] Sridhara SR, Paragache G, Panda NK, Chakrabarti A (2005). Mucormycosis in immunocompetent individuals: an increasing trend. *Journal of Otolaryngology*.

[3] Mallis A, Mastronikolis SN, Naxakis SS, Papadas AT (2010). Rhinocerebral mucormycosis: an update. *European Review for Medical and Pharmacological Sciences*.

[4] Zilberberg MD, Shorr AF, Huang H, et al (2014). Hospital days, hospitalization costs, and inpatient mortality among patients with mucormycosis: a retrospective analysis of US hospital discharge data. *BMC Infectious Diseases*.

[5] Skiada A, Pagano L, Groll A (2011). Zygomycosis in Europe: analysis of 230 cases accrued by the registry of the European Confederation of Medical Mycology (ECMM) working group on Zygomycosis between 2005 and 2007. *Clinical Microbiology and Infection*.

[6] Roden MM, Zaoutis TE, Buchanan WL (2005). Epidemiology and outcome of zygomycosis: a review of 929 reported cases. *Clinical Infectious Diseases*.

[7] Petrikkos G, Skiada A, Lortholary O, Roilides E, Walsh TJ, Kontoyiannis DP (2012). Epidemiology and clinical manifestations of mucormycosis. *Clinical Infectious Diseases*.

[8] Cornely OA, Arikan-Akdagli S, Dannaoui E (2014). ESCMID and ECMM joint clinical guidelines for the diagnosis and management of mucormycosis 2013. *Clinical Microbiology and Infection*.

[9] de Pauw B, Walsh TJ, Donnelly JP (2008). Revised definitions of invasive fungal disease from the European organization for research and treatment of cancer/invasive fungal infections cooperative group and the national institute of allergy and infectious diseases mycoses study group (EORTC/MSG) consensus group. *Clinical Infectious Diseases*.

[10] Petrikkos G, Skiada A, Sambatakou H (2003). Mucormycosis: ten- year experience at a tertiary-care center in Greece. *European Journal of Clinical Microbiology & Infectious Diseases*.

[11] Turunc T, Demiroglu YZ, Aliskan H, Colakoglu S, Arslan H (2008). Eleven cases of mucormycosis with atypical clinical manifestations in diabetic patients. *Diabetes Research and Clinical Practice*.

[12] Lanternier F, Dannaoui E, Morizot G (2012). A global analysis of mucormycosis in France: The RetroZygo study (2005–2007). *Clinical Infectious Diseases*.

[13] Ibrahim AS, Spellberg B, Walsh TJ, Kontoyiannis DP (2012). Pathogenesis of mucormycosis. *Clinical Infectious Diseases*.

[14] Gale GR, Welch AM (1961). Studies of opportunistic fungi. I. Inhibition of Rhizopus oryzae by human serum. *The American Journal of the Medical Sciences*.

[15] Peterson KL, Wang M, Canalis RF, Abemayor E (1997). Rhinocerebral mucormycosis: evolution of the disease and treatment options. *Laryngoscope*.

[16] Rüping MJ, Heinz WJ, Kindo AJ (2010). Forty-one recent cases of invasive zygomycosis from a global clinical registry. *The Journal of Antimicrobial Chemotherapy*.

[17] Rangel-Guerra RA, Martínez HR, Sáenz C (1996). Rhinocerebral and systemic mucormycosis. Clinical experience with 36 cases. *Journal of the Neurological Sciences*.

[18] Pagano L, Valentini CG, Posteraro B (2009). Zygomycosis in Italy: a survey of FIMUA-ECMM (Federazione Italiana di Micopatologia Umana ed Animale and European Confederation of Medical Mycology). *Journal of Chemotherapy*.

